# Diagnosis and Treatment of Suspected Osteomyelitis in a Complex Patient: A Case Report

**DOI:** 10.7759/cureus.111080

**Published:** 2026-06-18

**Authors:** Tyson Tetoff, Jasmin Kaur, Carlos Zambrano

**Affiliations:** 1 Medicine, Caribbean Medical University, Willemstad, CUW; 2 Medicine, Windsor University School of Medicine, Cayon, KNA; 3 Infectious Disease, Loretto Hospital, Chicago, USA

**Keywords:** bone infection, cefepime, chronic infection, osteomyelitis, vancomycin

## Abstract

Osteomyelitis is a devastating bone infection that occurs in individuals usually with significant comorbidities. This disease may lead to further major medical problems if not diagnosed and treated promptly. This case involves a 51-year-old male with multiple comorbidities, which led to the diagnosis of suspected chronic osteomyelitis. Laboratory work showed leukocytosis, decreased kidney function, anemia of unknown origin, and physical examination showing multiple skin ulcers. CT imaging supported the diagnosis of suspected chronic osteomyelitis. Due to the limited standard diagnostic testing modalities for osteomyelitis, significant clinical judgment had to be used and applied to treat osteomyelitis in this case. These diagnostic limitations included the absence of MRI evaluation, bone biopsy and subsequent histopathologic examination, as well as wound culture data. The antimicrobial drug use was influenced by the patient's underlying chronic kidney disease, which influenced treatment with cefepime and doxycycline rather than vancomycin. This case highlights the complexity of diagnosing and treating medically complex patients, with limited definitive diagnostic modalities, such as bone histopathology, wound cultures, and imaging, thus relying on clinical decision-making to formulate treatment options.

## Introduction

Osteomyelitis is a bone infection, often bacterial, commonly seen in intravenous (IV) drug users, immunosuppression, trauma/injury, or patients with chronic health conditions [1]. A patient may present with general symptoms consisting of fever, chills/sweating, fatigue, or nausea/vomiting, whereas local symptoms over the infected bone may include swelling, tenderness/pain, warmth, redness, joint stiffness, skin discoloration, or chronic back or musculoskeletal pain [2]. Acute osteomyelitis is acquired through hematogenous spread, often presenting clinically in the spine of the elderly or via direct inoculation with a systemic inflammatory response. In contrast, chronic osteomyelitis results from neglecting sequelae of acute osteomyelitis or from contiguous spread of non-healing pressure ulcers in individuals who may have limited mobility, such as paraplegics, patients who are bedridden, or patients who are diabetic with long-term foot problems (ulcers) [1,3]. The accepted definition of acute osteomyelitis is a bone infection that was acquired recently with a systemic response, but chronic osteomyelitis requires a minimal symptom duration of six weeks to three months [3]. Furthermore, surgeons classify osteomyelitis based on aspects of surgical approach, among which the Cierny-Mader classification is one of the most common [3]. 

The consideration of a diagnosis of osteomyelitis may be made in the patient after performing a detailed history and physical examination. Some “Red Flags” that potentially point towards an underlying diagnosis include, but are not limited to: immunosuppression, history of cancer, intravenous drug use, recent trauma, duration >6 weeks, prolonged glucocorticoid use, or history of osteoporosis. Possible differential diagnoses include fracture, septic arthritis, malignancy (e.g., Ewing sarcoma), gout, pseudogout, and sickle cell disease [2-4].

The treatment strategies for osteomyelitis require a multifaceted approach that varies among patients, including debridement of the surrounding tissue and/or infected bone, as well as a combination of intravenous and/or oral antibiotics depending on the clinical stage of the disease, as well as the culturing of the tissues, bone histology, and overall clinical viability of the patient [5]. Due to the inability of a bone biopsy and lack of culture and sensitivity, the treatment approach in this case was done from a bedside clinical matter and may be used to identify osteomyelitis in patients where there are limited clinical resources to perform the standard confirmatory tests. 

## Case presentation

A 51-year-old African American male presented to the emergency room with a complaint of chronic pain all over. The presenting history as well as past medical history are challenging, as the patient was confused on presentation to the ER. The patient looked chronically ill and irritable, appearing thin and poorly groomed. The patient stated he was diagnosed with right-sided lung cancer two years previously in another state and decided not to receive any treatment and did not follow up with any oncology service. The records could not be reviewed as access was limited. The patient stated he is bedbound and has multiple skin ulcers (Figures 1-3). Although the patient was unable to provide an accurate history regarding his living arrangement, he presented to the hospital from a nursing facility. Past medical history was significant for chronic hypertension with a left pontine cerebrovascular accident (CVA) as well as hyperlipidemia. Family history was significant for diabetes mellitus on his mother's side. This patient's past social history was positive for active tobacco use, smoking about half a pack of cigarettes daily, as well as marijuana use once weekly. The patient denied alcohol use.

**Figure 1:**
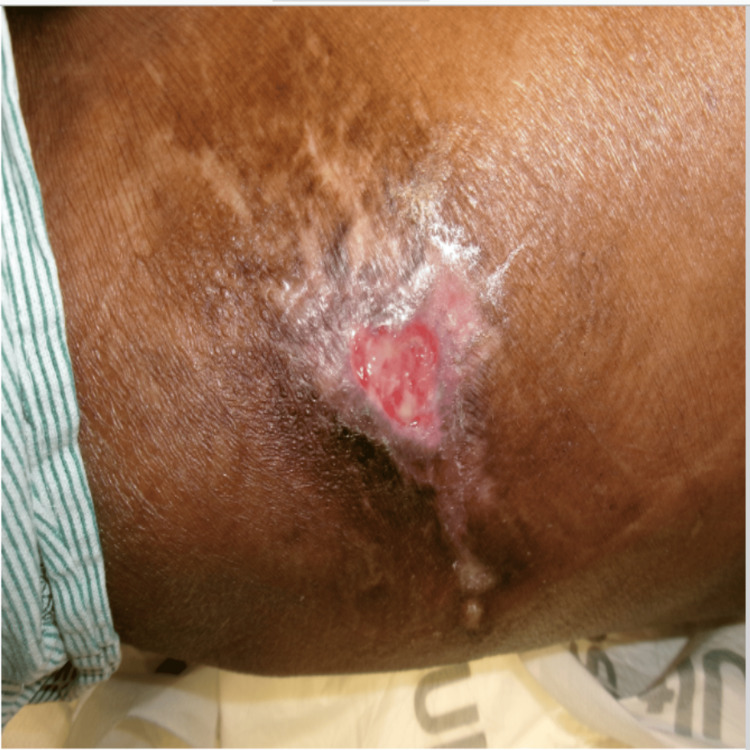
Skin ulcer on the posterior left thigh taken at admission to the hospital.

**Figure 2:**
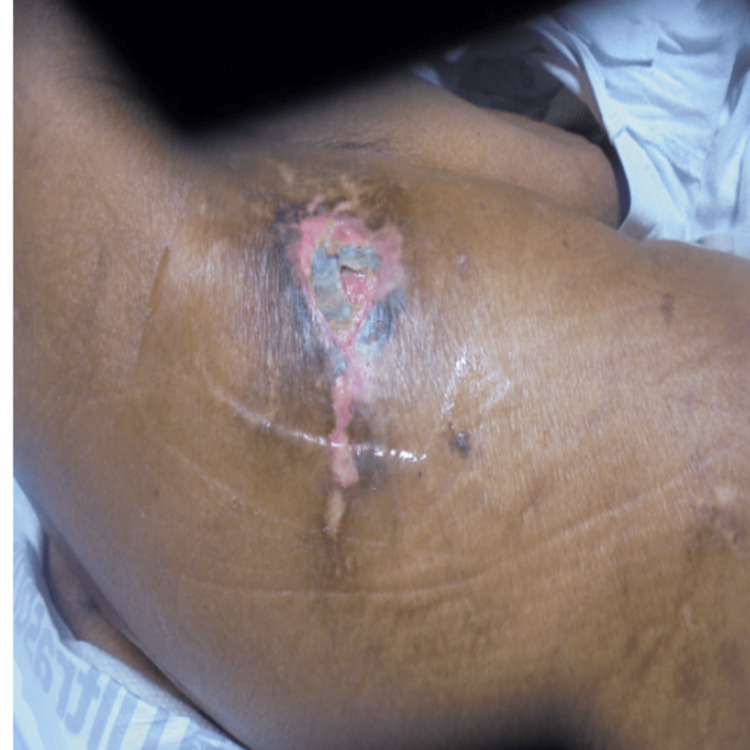
Skin ulcer on the left iliac crest taken at admission to the hospital.

**Figure 3:**
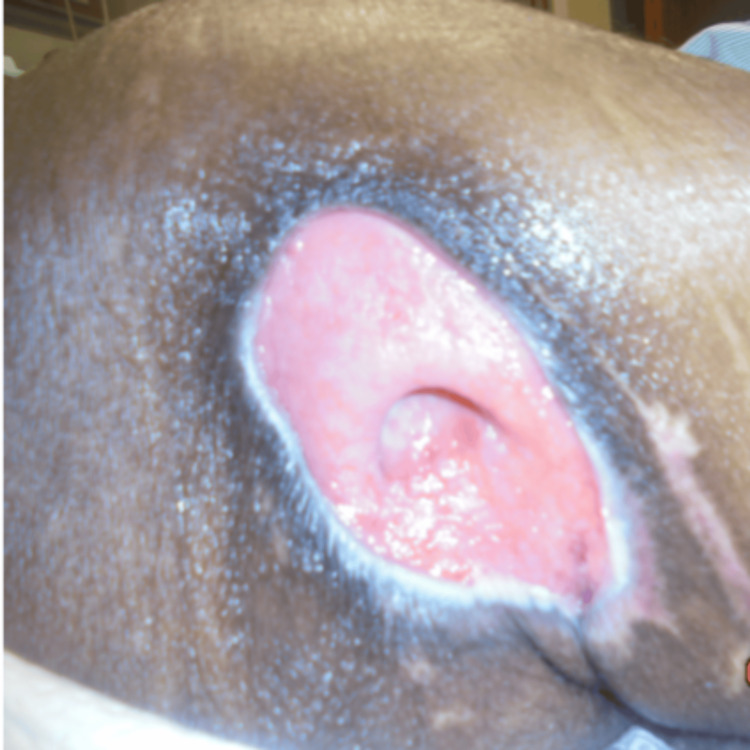
Posterior sacral decubitus ulcer with tunneling taken at admission to the hospital.

Initial physical evaluation showed poor dentation, poor respiratory effort, and right-sided CNS weakness, as well as multiple skin ulcers. A complete review of systems and physical examination had otherwise normal findings. Initial vital signs and significant laboratory values are shown in Table 1. Initial vitals showed a blood pressure of 106/64 mmHg, as shown in Table 1. Initial lab work presented acute kidney injury (AKI) with a blood urea nitrogen (BUN) 67 mg/dl, creatinine of 2.52 mg/dl and a reduced estimated glomerular filtration rate (eGFR) of 21.78 ml/min x 1.73 m^2^ (Table 1), in which the patient was placed on intravenous (IV) fluid replacement of 0.9% normal saline, and a nephrology consult was obtained. A complete blood count (CBC) demonstrated normocytic anemia, with a hemoglobin of 8.2 g/dl (Table 1). The patient was followed by primary care internal medicine and a hematology consult. Furthermore, due to the patient's wounds (Figures 1-3) and elevated white blood cell count (WBC) as seen on the CBC, an infectious disease consultation was obtained to evaluate the patient's infectious process and to assess the elevated WBC count. 

**Table 1:** Initial and final significant presenting vital signs and significant laboratory values from the presenting patient.

Parameters	Initial patient values (with units)	Final patient values (with units)	Reference range (with units)
Blood pressure	106/64 mmHg	147/60 mmHg	<120/<80 mmHg
Respiratory rate	10 breaths/min	18 breaths/min	12-20 breaths/min
White blood cells	11.3 x 10^3^ u/L	8.4 x 10^3^ u/L	4.0-11 x 10^3^ u/L
Hemoglobin	8.2 g/dL	8.9 g/dL	13.5-17.5 g/dL
Blood urea nitrogen (BUN)	67 mg/dL	89 mg/dL	8-23 mg/dL
Creatinine	2.52 mg/dL	1.77 mg/dL	0.5-1.1 mg/dL
Glomerular filtration rate	21.78 mL/min/1.73 m^2^	39.57 mL/min/1.73 m^2^	>90 mL/min/1.73 m^2^
Alpha 1 globulin	0.3 g/dl		0.2-0.4 g/dl
Alpha 2 globulin	0.8 g/dl		0.4-0.8 g/dl
Beta 1 globulin	0.4 g/dl		0.3-0.6 g/dl
Beta 2 globulin	0.2 g/dl		0.2-0.5 g/dl
Gamma globulin	0.9 g/dl		0.6-1.7 g/dl
Kappa	242 mg/dl		176-443 mg/dl

Due to the presence of the patient's chronic skin ulcers (Figures 1-3), specifically, the presence of a Grade III sacral pressure ulcer with tunneling and tan exudate and a right-sided Grade IV trochanteric pressure ulcer with tan exudate drainage, this raised concerns for an underlying soft tissue infection and possible osteomyelitis. Consultations with wound care as well as infectious disease were obtained for further evaluation and management.

An initial dose of intravenous vancomycin 1 gram was given on presentation in the emergency room before admittance to the hospital ward. Due to the reduced eGFR and elevated BUN and creatinine levels, the decision was made to stop IV vancomycin. The patient was then started on a scheduled dose of intravenous cefepime 1 gram every 12 hours until the confirmed diagnosis could be made. On day 10 of admission, doxycycline 100 mg orally twice a day was added for broad-spectrum coverage of suspected skin pathogens. 

On day 10 of admission, an MRI was attempted to assess and evaluate for suspected osteomyelitis. This procedure could not be completed and was aborted because the patient was unable to remain motionless during the scan; therefore, no adequate images were obtained. On day 11 of admission, an abdominal CT without contrast was performed in lieu of the MRI and showed mixed lytic and sclerotic bone destruction of the left iliac crest, sacral decubitus ulceration, and a tiny air bubble adjacent to the right greater trochanter of the femur (Figures 4-7). The possible diagnosis of multiple myeloma was also considered due to the patient's previous history of cancer without treatment and the finding of lytic bone lesions on the CT scan. Alpha-, beta-, gamma-, kappa-, and lambda protein electrophoresis was performed, with all results being returned and falling within normal parameter ranges, as shown in Table 1, and was reviewed by the hematologist.

**Figure 4:**
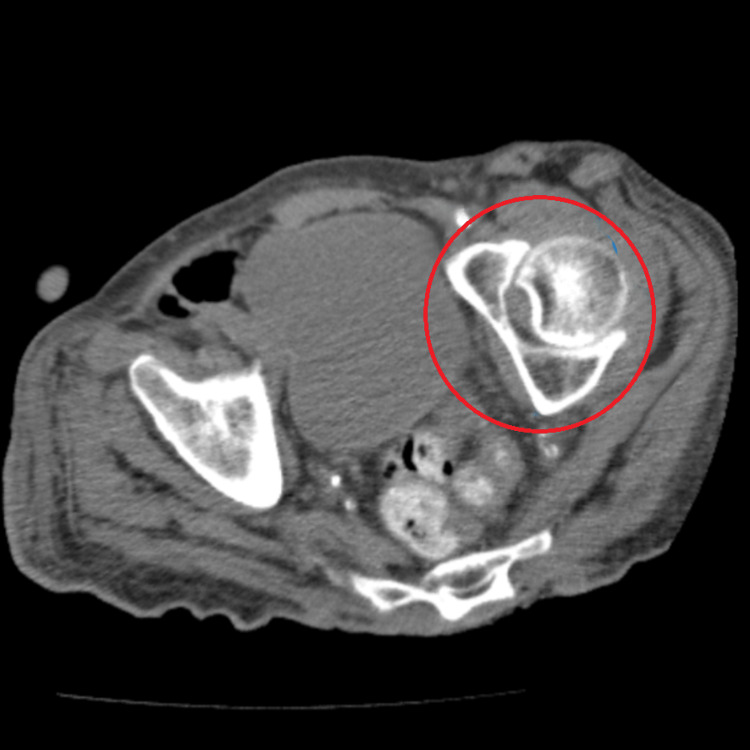
CT image without contrast showing lytic and sclerotic bone destruction of the left iliac crest, as shown by the lightened and darkened areas in the bone.

**Figure 5:**
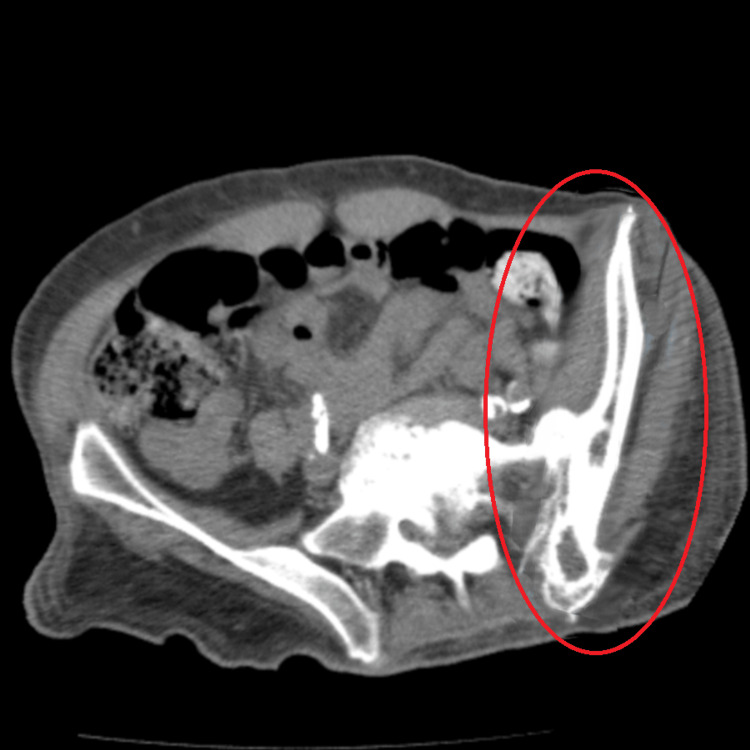
CT image without contrast showing lytic and sclerotic bone destruction of the left iliac crest, as shown by the lightened and darkened areas in the bone.

**Figure 6:**
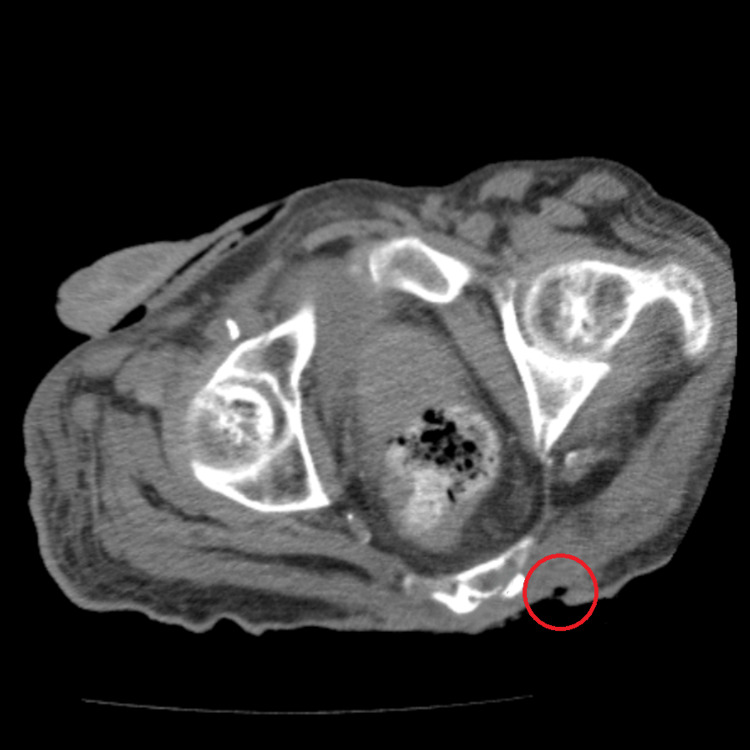
CT image without contrast showing a Grade III sacral decubitus ulcer with tunneling on the lower left side of the skin tissue.

**Figure 7:**
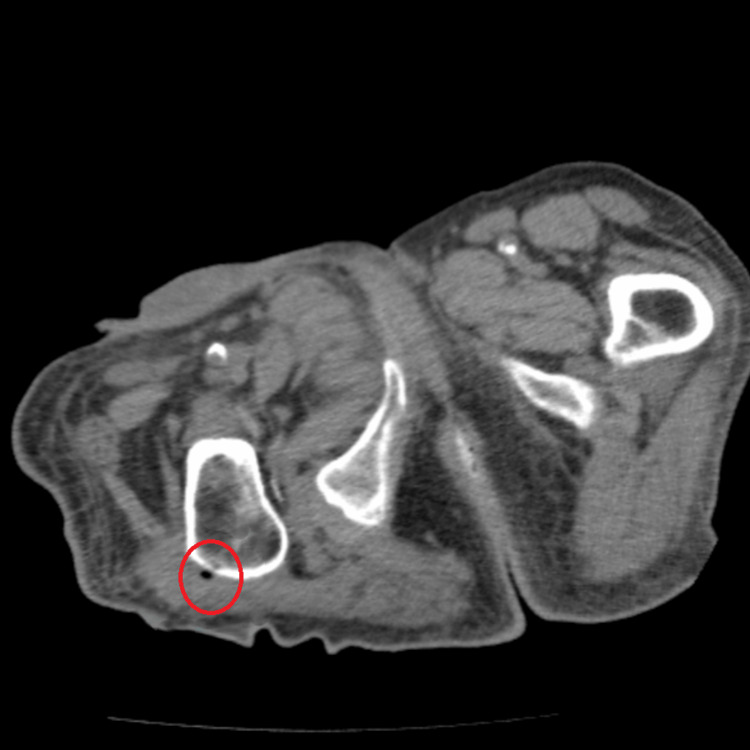
CT image without contrast showing a small air bubble posterior to the right greater trochanter.

With multiple myeloma ruled out, the positive findings on the CT scan of a small air bubble, along with clinical correlation of symptoms of pain, lack of range of motion, and history of skin ulcers, the suspected diagnosis of chronic osteomyelitis was determined by the infectious disease consultation. Due to lack of hospital resources and logistics, a confirmatory bone biopsy was unable to be obtained. Wound swabs for bacterial growth cultures and antibiotic sensitivity were also not performed on the skin ulcers. The patient was continued on antibiotic treatment of cefepime and doxycycline as an inpatient for six weeks under the care of multiple medical teams. 

## Discussion

Osteomyelitis is defined as either an acute or chronic secondary infectious inflammation of the bone due to a primary infectious process [5]. The differentiation between acute and chronic osteomyelitis is not based on the duration of the infection but on histopathological findings [6]. Histopathological findings in acute osteomyelitis are defined by micro-organisms, thromboses, or congested blood vessels and infiltrates showing neutrophils, whereas the chronic histopathological defining features are necrotic bone [7]. Furthermore, acute osteomyelitis infection occurs prior to the development of sequestra and typically happens within two weeks of onset, whereas the development of sequestra and necrotic bone classifies osteomyelitis as chronic [7]. 

The incidence of osteomyelitis in the USA is hard to determine and is mostly not known, but has been reported as approximately 50,000 cases per year or about 1/675 USA hospital admissions [8]. A population-based study from 1969 to 2009 showed that the incidence remained stable among children and young adults but almost tripled in individuals who were older than 60 years old [9]. Furthermore, this study showed that although the incidence was higher in men, for unknown factors, it is theorized to be due to the increased prevalence of co-morbid factors [8,9]. 

Osteomyelitis is most commonly or typically diagnosed via MRI with contrast, as it is the most sensitive, having a sensitivity of 78 to 90%, and a non-invasive imaging modality [5]. Other diagnostic imaging includes plain radiography, technetium-99 bone scan, CT, and PET scan [5]. As osteomyelitis progresses, the initial osteolytic areas become surrounded by sclerotic bone with ongoing repair and remodeling, which may lead to osteosclerosis and hyperostosis [10]. The prognosis of osteomyelitis varies from patient to patient. Most individuals recover without long-term complications if diagnosed early and the treatment regimen is started promptly. 

There are several different antibiotic and/or surgical treatment options of osteomyelitis along with alternative regimens for patients with concurrent diagnoses [5]. General treatment therapies, as well as the organisms that are covered by the antibiotics of choice, are long-established and are proven [5]. Long-term antibiotic therapy, as well as surgical debridement of the affected area, is the standard most effective way to treat osteomyelitis [7]. The most commonly used antibiotic regimen is vancomycin (to cover gram-positive as well as methicillin-resistant *Staphylococcus aureus* (MRSA)) and a cephalosporin or beta-lactamase inhibitor (for gram-negative as well as gram-positive coverage) [5,7]. There are many algorithms and published pathogen-specific antibiotic therapies to treat the differing organisms, as well as antibiotics to treat the pathogens that cause osteomyelitis. Although cultures and bone biopsy were not obtained in this patient, the CT scan findings and the bedside presentation suggested chronic osteomyelitis, which was confirmed by the infectious disease consultation, and the antibiotic regimen was administered to treat the suspected pathogens.

Upon presentation and before confirmation of cultures with sensitivity could be obtained, the decision was made on review of initial white blood cell count as well as clinical examination, that an initial dose of prophylactic vancomycin 1 gram intravenously was to be administered. Upon further evaluation of the kidney function of the patient, it was determined to stop the vancomycin treatment. This was done due to the patient's BUN and creatinine being elevated, as well as eGFR being reduced. This is due to vancomycin's elimination from the body being approximately 80-90% renal with a decreasing linear relationship to creatinine clearance [11]. Antibiotic therapy was then tailored to a patient with CKD. Although cefepime is also eliminated approximately 85% renal [12], the molecular size of cefepime at 480.6 g/mol compared to that of vancomycin at 1449.2 g/mol [13,14]. Owing to vancomycin's large molecular size and clearance via renal elimination, the drug's clearance is markedly reduced in this patient with CKD and thus was not the antibiotic of choice, as these factors may lead to drug accumulation and nephrotoxicity. 

Furthermore, the lytic and sclerotic bone lesions that were seen must be further investigated. Patients with a non-small-cell lung cancer diagnosis generally have metastases outside the thoracic cavity, spreading to the adrenal glands, liver, bones, brain, and lymph nodes [14]. The pathophysiology of osteolytic and osteosclerotic lesions is a complex mechanism that uses many differing chemokines, ligands, and growth factors [15]. This must be investigated further by oncological services if the patient allows, as it is noted that the patient previously denied treatment of his right-sided lung cancer two years prior.

## Conclusions

Several predisposing factors increased the suspected diagnosis of osteomyelitis, such as multiple skin ulcers, chronic tobacco use, and the CT imaging showing a Grade III tunneling ulcer and an air bubble on the posterior right trochanter, as well as the mixed sclerotic and lytic bone destruction in the left iliac crest. The clinical presentation, in conjunction with the radiographic findings, raised significant suspicion for the diagnosis of osteomyelitis despite the absence of standard definitive diagnostic testing. The inpatient treatment for the suspected osteomyelitis consisted of doxycycline 100 mg orally twice a day for treatment of the skin ulcers/musculoskeletal infection and cefepime 1 gram intravenously every 12 hours for extended-spectrum coverage for gram-negative and gram-positive bacteria. This antibiotic treatment was planned for a six-week duration.

The patient expressed a desire to leave the hospital as soon as possible. Although his blood pressure improved and leukocytosis resolved, a complete clinical resolution of the suspected osteomyelitis could not be fully assessed due to the lack of definitive culture data and advanced imaging. Upon hospital discharge, the patient was prescribed oral cefprozil 500 mg orally every 12 hours and minocycline 100 mg orally every 12 hours for an additional 14 days. The patient was also instructed to follow up with wound care services as an outpatient for treatment of the musculoskeletal wounds, nephrology as an outpatient for the non-improving AKI and to have MRI imaging done within two to three weeks after discharge to monitor changes of the lytic and sclerotic bone destruction. 

This case highlights the challenges associated with diagnosing and managing suspected osteomyelitis in a medically complex patient within a resource-limited healthcare setting. The inability to obtain a bone biopsy and MRI imaging requires clinicians to rely on clinical judgment, other radiographic findings, and laboratory data to establish a diagnosis and formulate an appropriate treatment plan. This case underscores the importance of integrating available clinical and diagnostic information when definitive testing is inaccessible and emphasizing the need for close outpatient follow-up and continued evaluation.

## References

[1] Kronig I, Vaudaux P, Suvà D (2015). Acute and chronic osteomyelitis. Clinical Infectious Disease.

[2] (2026). Cleveland Clinic: Osteomyelitis (bone infection). https://my.clevelandclinic.org/health/diseases/osteomyelitis-bone-infection.

[3] Ali AM, Khan M, Kwatra SG, Barbaryan A, Hussain N, Saba R, Mirrakhimov AE (2013). Unusual case of osteomyelitis and discitis in a drug user with a background of chronic back pain: do not miss the serious etiologies. Case Rep Infect Dis.

[4] 4) Patel, D. (2025, July 28) (2026). Medscape: Osteomyelitis differential diagnoses. https://emedicine.medscape.com/article/1348767-differential.

[5] Bury DC, Rogers TS, Dickman MM (2021). Osteomyelitis: diagnosis and treatment. Am Fam Physician.

[6] Hatzenbuehler J, Pulling TJ (2011). Diagnosis and management of osteomyelitis. Am Fam Physician.

[7] Nimmana BK, Savaliya V (2023). Osteomyelitis. StatPearls (Internet).

[8] Rubin RJ, Harrington CA, Poon A, Dietrich K, Greene JA, Moiduddin A (1999). The economic impact of Staphylococcus aureus infection in New York City hospitals. Emerg Infect Dis.

[9] Kremers HM, Nwojo ME, Ransom JE, Wood-Wentz CM, Melton LJ III, Huddleston PM III (2015). Trends in the epidemiology of osteomyelitis: a population-based study, 1969 to 2009. J Bone Joint Surg Am.

[10] Kang HJ, Choi HY, Park JS (2018). Lesions that mimic musculoskeletal infection: a pictorial essay. J Korean Soc Radiol.

[11] Vandecasteele SJ, De Vriese AS (2010). Recent changes in vancomycin use in renal failure. Kidney Int.

[12] Pais GM, Chang J, Barreto EF (2022). Clinical pharmacokinetics and pharmacodynamics of cefepime. Clin Pharmacokinet.

[13] (2026). PubChem: Vancomycin. https://pubchem.ncbi.nlm.nih.gov/compound/14969.

[14] (2026). PubChem: Cefepime. https://pubchem.ncbi.nlm.nih.gov/compound/cefepime.

[15] Ali Mohammed Hammamy R, Farooqui K, Ghadban W (2018). Sclerotic bone metastasis in pulmonary adenocarcinoma. Case Rep Med.

